# N6-methyladenosine regulates ATM expression and downstream signaling

**DOI:** 10.7150/jca.64061

**Published:** 2021-10-17

**Authors:** Xiaoyue Zhang, Peishan Liu, Xiang Zheng, Jia Wang, Qiu Peng, Zhengshuo Li, Lingyu Wei, Can Liu, Yangge Wu, Yuqing Wen, Qun Yan, Jian Ma

**Affiliations:** 1 Hunan Cancer Hospital and the Affiliated Cancer Hospital of Xiangya School of Medicine, Central South University, Changsha, China.; 2 Cancer Research Institute and School of Basic Medical Science, Central South University, Changsha, China.; 3 Key Laboratory of Carcinogenesis and Cancer Invasion of the Chinese Ministry of Education, NHC Key Laboratory of Carcinogenesis, Hunan Key Laboratory of Nonresolving Inflammation and Cancer, Hunan Key Laboratory of Cancer Metabolism, Changsha, China.; 4 Department of Pathology, Affiliated Hospital of Guilin Medical University, Guilin, Guangxi, China.; 5 Department of Immunology, Department of Pathology, Heping Hospital, Changzhi Medical College, Changzhi, Shanxi, China.; 6 Department of Clinical Laboratory, Xiangya Hospital, Central South University, Changsha, China.

**Keywords:** m6A modification, ATM, DNA damage response, METTL3, YTHDF1

## Abstract

N6-methyladenosine (m6A) is the most abundant modification in eukaryotic mRNAs, which plays an important role in regulating multiple biological processes. ATM is a major protein kinase that regulates the DNA damage response. Here, we identified that *ATM* is a m6A-modificated gene. METTL3 (a m6A “writer”) and FTO (a m6A “eraser”) oppositely regulated ATM expression and its downstream signaling. Mechanically, m6A “readers” YTHDFs and eIF3A suppressed ATM expression in the post-transcriptional levels. We also revealed the oncogenic potential of METTL3 and YTHDF1 related to ATM modulation. This is the first report that ATM, a master in the DNA damage response, is modified by m6A epigenetic modification, and METTL3 disrupts the ATM stability via m6A modification, thereby affecting the DNA-damage response.

## Introduction

N6-methyladenosine (m6A) is the most prevalent and representative chemical modification in eukaryotic mRNAs as well as other non-coding RNAs, which are widely found in yeast, Arabidopsis, Drosophila, mammals, and viruses 1, 2. m6A modification system is composed of “writers”, “erasers”, and “readers”, which allows the m6A modification becoming a dynamic and reversible regulatory process. METTL3 is the major catalytic subunit and was the first identified methyltransferase (“writer”), while METTL14, as another “writer”, plays a crucial role in substrate recognition 3, 4. METTL3 and METTL14 proteins can form a stable heterodimeric core complex that promotes m6A deposition 5, 6. m6A “erasers” are demethyltransferases, which erase m6A modification and play reversible regulatory roles, mainly including FTO and ALKBH5 7-9. m6A “readers” specifically target m6A sites in a methylation-dependent manner, which mainly include YTHDF1-3, eIF3, nuclear protein YTHDC1-2, and other molecules 10-12. Different “readers” bind to the m6A modified sites and function as RNA-binding proteins in many biological processes. YTHDF1 recognizes m6A-modified mRNA that results in decreased translation efficiency 13, whereas YTHDF2 reduces mRNA stability with recruitment of mRNA degradation systems 14. YTHDF3 cooperates with YTHDF1 to promote protein synthesis and mediates the decay of methylated mRNA with the help from YTHDF2 15.

ATM is a protein kinase, a major molecule that regulates the DNA damage response by phosphorylating a variety of key proteins in response to DNA double-strand breaks and DNA single-strand breaks, thus playing a key role in DNA damage repair 16. In higher eukaryotes, ATM kinase and ATR kinase are two important kinases governing the DNA damage response. ATM and ATR belong to the PI3K-like kinase (PIKK) family along with the DNA-dependent protein kinase catalytic subunits (DNA-PKcs) encoded by the *PRKDC* gene 17. ATM is a component of multiple signaling pathways, including cell growth 18, metabolism 19, protein synthesis, and autophagy 20, which are involved in tumorigenesis. For instance, ATM-mediated phosphorylation activates and stabilizes the tumor suppressor p53, which triggers cell cycle arrest by upregulating the cell cycle protein-dependent kinase inhibitor p21, resulting in induction of apoptosis 21.

Notably, a stressful m6A modification of intracellular RNA was found to occur following only 2 minutes of UV irradiation. This modification can occur on a variety of poly(A)+ transcripts and is regulated by METTL3 and FTO. UV light induces the formation of cyclobutane pyrimidine dimer adducts on the genome, and repair against this adduct is delayed when cells lack METTL3 catalytic activity 22. This study demonstrates the importance of m6A modifications in the DNA damage response. However, the presence of m6A modification in *ATM* mRNA, and its corresponding regulatory mechanisms have not been reported.

Previous studies 23-25 including ours 26 have identified the effect of Epstein-Barr Virus (EBV) infection on the level of transcriptomic m6A modifications in host cells. Upon EBV infection, we identified increased levels of m6A modification of *ATM* mRNA. In the current study we revealed a potential role of m6A modification in ATM expression and the downstream signaling in the DNA damage response. Our study illustrates a novel regulatory mechanism of DNA damage response through m6A modification of the *ATM* mRNA.

## Materials and methods

### Cell culture and chemicals

BJAB (EBV-negative B lymphoma cell line) and Raji (EBV-positive B lymphoma cell line) cells were maintained in PRMI-1640 (Hyclone) supplemented with 10% fetal bovine serum (FBS). SGC7901 (gastric cancer cell line) and HEK293 cells were cultured in DMEM medium (Gibco) supplemented with 10% FBS. All cell lines were obtained from the American Type Culture Collection (ATCC). The cell lines were tested negative for mycoplasma contamination. Cycloheximide (CHX, M4879) and MG132 (M1902) were purchased from AbMole (Houston, USA). 3-deazaadenosine (3-DAA) was purchased from Sigma-Aldrich.

### Cell transfection, plasmids and RNA interference

Cells were cultured to a predetermined time. The siRNAs and plasmids were transfected using Lipofectamine 3000 (Invitrogen) according to the manufacturer's instruction. DNA fragments encoding full-length ORF of YTHDF1, YTHDF2, or YTHDF3 were cloned into a Flag-tagged pcDNA3.1 (+) empty vector. shRNA lentiviruses and siRNA (targeting METTL3, YTHDF1, YTHDF2, or YTHDF3) were obtained from GenePharma (Shanghai, China). Lentiviruses were transduced into cells according to the manufacturer's instructions. The siRNA and shRNA sequences are listed in Table S1.

### MeRIP-qPCR

According to the manufacturer's protocol, intact poly-A-purified RNA was isolated using Magnetic mRNA Isolation Kit (S1550S, New England Biolabs). With reverse transcription with Oligo(dT) and PCR amplification, mRNA incubated with 5 μg m6A antibody (202003; Synaptic Systems) in IP buffer (PH7.4) containing RNase inhibitor (N2111SV, Promega), 150 mM NaCl, 10 mM Tris-HCl and 0.1%NP-40 for 2 h at 4 °C. Protein A/G magnetic beads (823202, Selleck) were washed, incubated with mixture buffer for 2 h at 4 °C with rotation. m6A RNA was eluted with 6.7 mM m6A sodium salt (Santa Cruz Biotechnology) and precipitated with 100% ethanol. Enrichment of m6A was detected through qRT-PCR. Normal rabbit IgG were designed as negative control in this experiment group.

### RNA immunoprecipitation (RIP) assays

Briefly, Cells were transfected with Flag-YTHDF1 or Flag-YTHDF2 or Flag-YTHDF3 plasmids, respectively, for 48 h. Then cells were harvested in RIP lysis buffer including RNase inhibitor (N2111SV, Promega), protease inhibitor and phosphatase inhibitor (b14001; b15001, Selleck). The supernatants were retained and mixed with anti-Flag antibody (F1804, Sigma-Aldrich) or normal mouse anti-IgG (sc-2025; Santa Cruz Biotechnology) for 12 h at 4 °C after centrifugation with 12000 rpm at 4 °C. Protein A/G magnetic beads were added to the mixture and incubated for 4 h at 4 °C with rotation. The beads were then washed with RIP wash buffer. Total RNA was isolated and proved to qRT-PCR analysis.

### RNA isolation and reverse transcription (RT)-PCR assay

Total RNAs from cells were isolated using TRIzol reagent (Invitrogen). 2 μg RNA was reverse transcribed into synthesize complementary DNA (cDNA) using RevertAid RT Reverse Transcription Kit (Thermo Scientific). Real-time reverse-transcription PCR was carried out by SYBR premix Ex TaqII Kit (Takara). Actin was performed in parallel as a control. Each mRNA expression was quantified by measuring cycle threshold (Ct) values. The relative expressions were calculated using the 2^-ΔΔCt^ method. The primers for qRT-PCR are listed in Table S2.

### Western blot

Protein extracts were resolved by sodium dodecyl sulfate-polyacrylamide gel, transferred to polyvinylidene fluoride membranes, and probed with antibodies against ATM (2873S, Cell Signaling Technology, CST); p-ATM -S1981 (AP0008, ABclonal); METTL3 (15073-1-AP, Proteintech); YTHDF1 (17479-1-AP, Proteintech); YTHDF2 (24744-1-AP, Proteintech); YTHDF3 (25537-1-AP, Proteintech); FTO (TA809392, OriGene); m6A (202003, Synaptic Systems); BRCA1 (20649-1-AP, Proteintech); H2A.X (A11361, ABclonal); γH2A.X (AP0687, ABclonal ); GAPDH(60004-1-Ig, Proteintech); eIF3A (3411T, CST); Flag (F1804, Sigma-Aldrich); anti‐rabbit IgG HRP‐linked antibody (7074; CST); Peroxidase-conjugated Affinipure Goat Anti-Mouse IgG (H+L) (SA00001-1, Proteintech); α-Tubulin (66031-1-Ig, Proteintech); CHK1(bs-1681R, Bioss); CHK2 (252092, Zenbio); p-CHK2-S19(AP0862, ABclonal); Anti-HA tag monoclonal antibody (TA100012, OriGene); Mouse-IgG (BA1046, Boster); Rabbit IgG (B900610, Proteintech); p21(10355-1-AP, Proteintech); 53BP1 (BA2878, Boster); CDK1 (D160158, BBI life sciences); CDK2 (D199431, BBI life sciences); Cyclin B2 (21644-1-AP, Proteintech).

### Cell cycle assay

SGC7901 cells were transfected with siRNA and incubated until a predetermined time. Wash the cells twice with pre-chilled PBS, collect the cells by centrifugation and discard the supernatant, add 50 μL of Binding Buffer and gently resuspend the cell precipitate. Add 1 mL of 70% pre-cooled ethanol to fix the cells and place the cells in the rotator overnight at 4 °C. Centrifuge at 400 g for 5 min at 4 °C, wash the cells twice with PBS, centrifuge, and discard the PBS. Add staining solution and stain the cells at 37 °C for 30 min under low light and perform flow cytometry assay. The whole procedure should be as light as possible to avoid cell fragmentation.

### Co-Immunoprecipitation (Co-IP) assay

Cells were transfected with indicated plasmids and cultured to a predetermined time, cells were lysed on ice with IP lysis solution, cell supernatant was collected and mixed with anti-Flag antibody or anti-IgG antibody (negative control), and rotated overnight at 4 °C. Protein A/G magnetic beads were added, and the mix was incubated for 2 h at 4 °C. The magnetic beads were adsorbed on a magnetic frame, the supernatant was discarded, and Western blot experiments were performed.

### Immunofluorescence assay

Cells were transfected with siRNAs for 48 h, and then were fixed in medium containing 4% paraformaldehyde for 1 h and then permeabilized using 0.5% Triton X-100 and blocked using normal goat serum. The primary antibodies (anti-p-ATM, or anti-γH2A.X) were added and incubated at room temperature for 2 h. Secondary antibody (Cy3 conjugated; red) (Beyotime) were added and incubated for 1 h. Stained cells were examined using a fluorescence microscope.

### RNA stability assay

Cells were added with actinomycin-D (Act-D, Meilunbio) at 5 μg/ml to measure RNA stability in cells with siRNA or shRNA pre-treatment. Cells were harvested at an indicated time points and extracted RNA for reverse transcription. The mRNA transcript levels were normalized to 0 h of actinomycin-D treatment by RT-qPCR.

### CCK8 cell proliferation assay

Cells were transfected with siRNA culturing in 96-well plates (1000 cells per well). Cells were cultured until a predetermined time in the presence or absence of the ATM inhibitor KU55933 (10 μM). 10 μL CCK8 reagents (CK04, Dojindo) were added in 96-well plates incubating for 4 h at 37 °C. Absorbance was obtained at 450 nm by a microplate reader (ThermoFisher).

### Statistical analysis

Statistical significance was calculated using Prism (GraphPad Software) and SPSS17. All experiments were performed in triplicate. Data represent the mean ± SD. Statistical differences were assessed with the unpaired Student t-test, and p <0.05 were considered to reflect statistical significance. A one‐way analysis of variance test was performed for comparing three or more groups within the same experiment.

## Results

### Identification of *ATM* as an m6A-modificated gene

**Figure 1:**
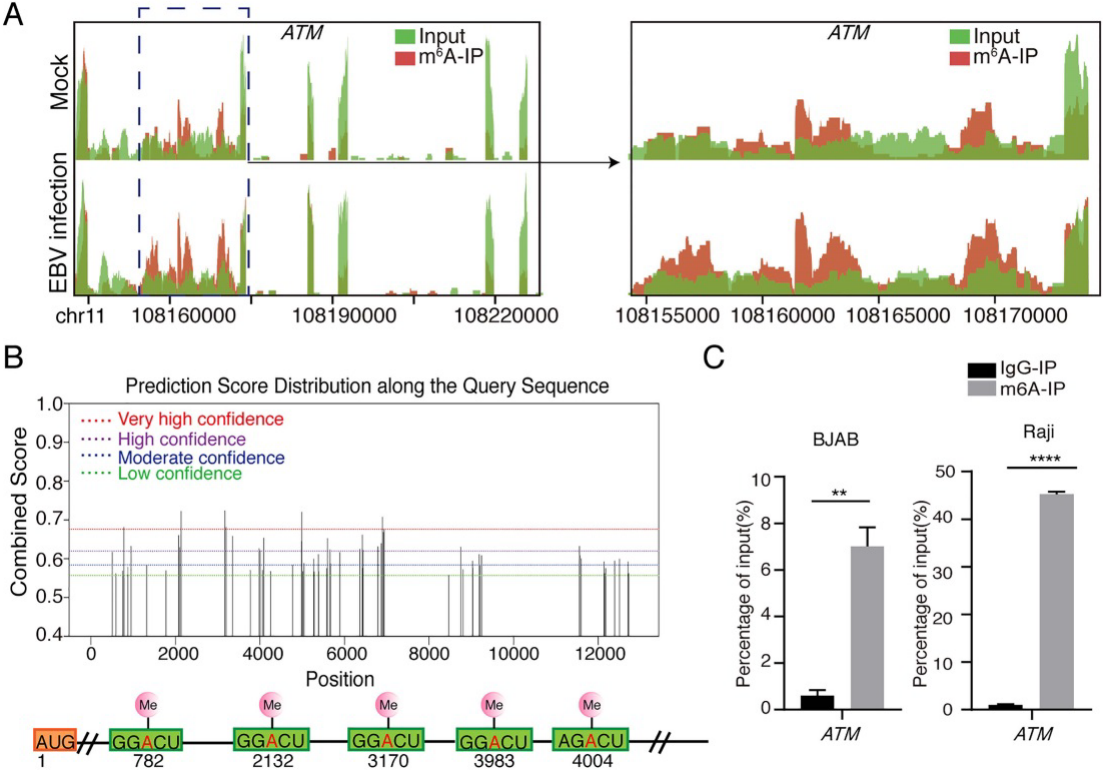
** Presence of m6A modification in *ATM* mRNA. (A)** MeRIP‐seq of BJAB cells which were infected by EBV (or uninfected as a negative control) for 24 h. Visualized images show the enrichment of m6A immunoprecipitation (red) over input (green) on the *ATM* mRNA fraction region. **(B)** Predictions of potential m6A modification sites for *ATM* mRNA sequences from the SRAMP website. **(C)** BJAB or Raji cells were cultured to a predetermined time, mRNA was enriched from cell extracted RNA and immunoprecipitated with anti-m6A antibody (or IgG antibody as negative control). mRNA eluted from the immunoprecipitation was quantified as a percentage of input. RT-qPCR was performed to detect the expression levels of *ATM*. Values are the mean ± SD (n = 3). Two-tailed unpaired t test. **p < 0.01, ****p < 0.0001 compared with control group.

From our previous results 26, we found that the m6A abundance was increased in *ATM* mRNA transcripts upon EBV infection (Figure 1A). ATM is a key kinase in DNA damage response, which is responsible for detecting, transmitting and repairing DNA damage response 21. However, the role of the m6A modification involved in ATM signaling is not yet fully understood. SRAMP (sequence-based RNA adenosine methylation site predictor) 27, a bioinformatical tool to predict the m6A site of mRNAs, has predicted five high confidence m6A sites in *ATM* mRNA sequences (Figure 1B). MeRIP-RT-qRCR assay was performed to detect enriched *ATM* mRNAs after anti-m6A immunoprecipitation. *ATM* mRNAs were specifically enriched by the anti-m6A antibody in cells (Figure 1C). These results suggested that *ATM* gene is m6A modified in cells.

### METTL3 and FTO modulate ATM expression

**Figure 2:**
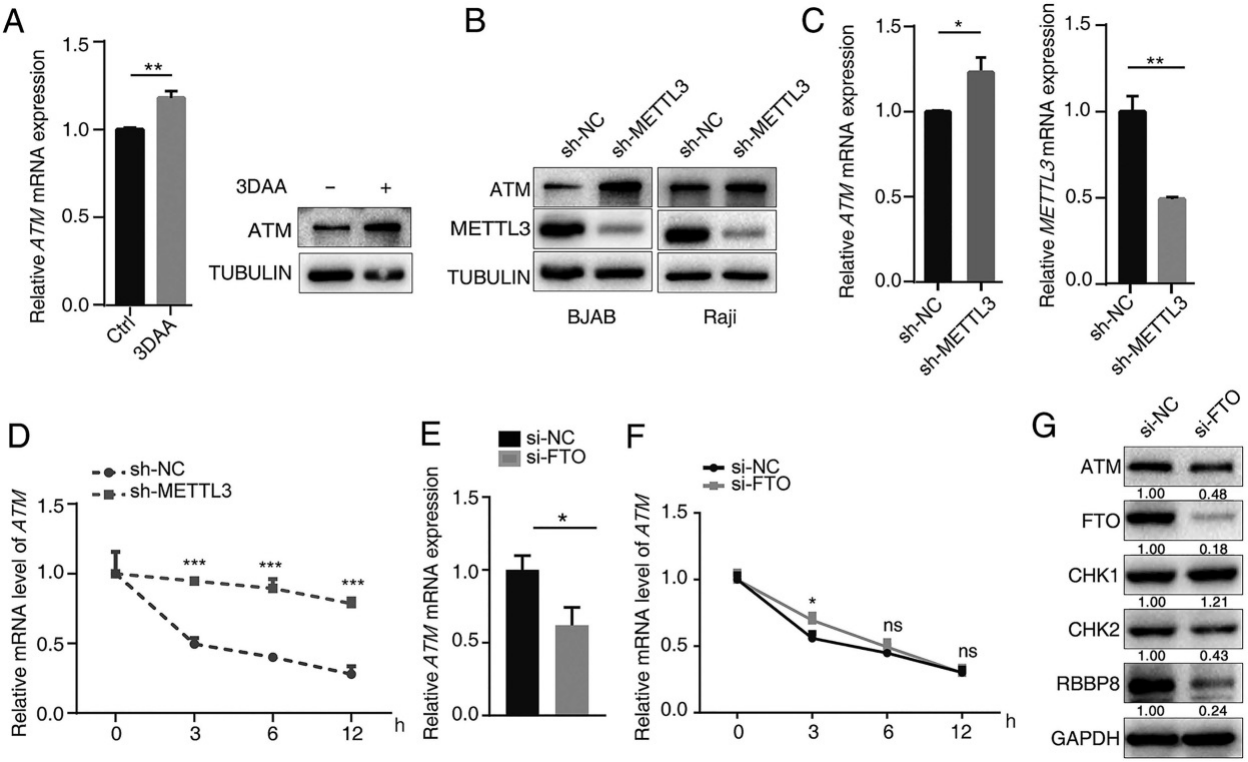
** METTL3 and FTO modulate ATM expression. (A)** BJAB cells were treated with 10 μM 3-DAA (control group were treated with equal amount of PBS) for 2 h, cellular RNA was extracted at 24 h and mRNA expression of *ATM* was assayed by RT-qPCR (n=3). Western blot assay was used to detect ATM protein expression. **(B)** Western blot assay was used to detect ATM and METTL3 protein expression levels in sh-NC or sh-METTL3 cells. **(C)** RT-qPCR was used to detect *ATM* and *METTL3* mRNA expression levels in sh-NC or sh-METTL3 BJAB cells. **(D)** BJAB cells (sh-NC and sh-METTL3) were treated with Act-D (5 μg/ml) for indicated times, then, RT-qPCR assay was performed to detect *ATM* mRNA expressions. Mean ± SD of three independent experiments. Two-tailed unpaired t-test. **(E)** SGC7901 cells were transfected with indicated siRNAs for 24 h. *ATM* mRNA expression was detected by RT-qPCR. **(F)** SGC7901 cells (si-NC and si-FTO) were treated with Act-D (5 μg/ml) for indicated times, then, RT-qPCR assay was performed to detect *ATM* mRNA expressions. **(G)** SGC7901 cells were treated with indicated siRNAs for 48 h, Western blot was used to detect the indicated proteins. *p < 0.05, **p < 0.01, ***p < 0.001. ns, no significant difference.

3-deazaadenosine (DAA) is a general methylation inhibitor, and we found that it can substantially increase the ATM mRNA and protein levels in cells (Figure 2A), suggesting methylation levels contributed to ATM expression regulation. Since METTL3 is the main m6A “writer”, we used shRNA to knockdown METTL3 expression in cells, and found that silence of METTL3 resulted in significant increase in ATM expression (Figure 2B, C). sh-METTL3 delayed the degradation of *ATM* mRNA (Figure 2D), indicating that METTL3 inhibited *ATM* expression and decreased its mRNA stability. FTO is a demethylase for m6A modification, and eliminates m6A modifications on mRNAs 28. Inhibition of endogenous FTO significantly reduced ATM expression levels (Figure 2E, G). However, inhibition of FTO did not affect *ATM* mRNA stability (Figure 2F). In addition, we examined whether the downstream signaling of ATM were regulated by FTO. Western blot analysis revealed that inhibition of FTO decreased the expression levels of ATM, CHK2, and RBBP8 (Figure 2G). These results suggested METTL3 (“writer”) and FTO (“eraser”) could regulate ATM expression and its downstream signaling.

### METTL3 promotes cell cycle progression, cell proliferation and involves in the DNA damage response

**Figure 3:**
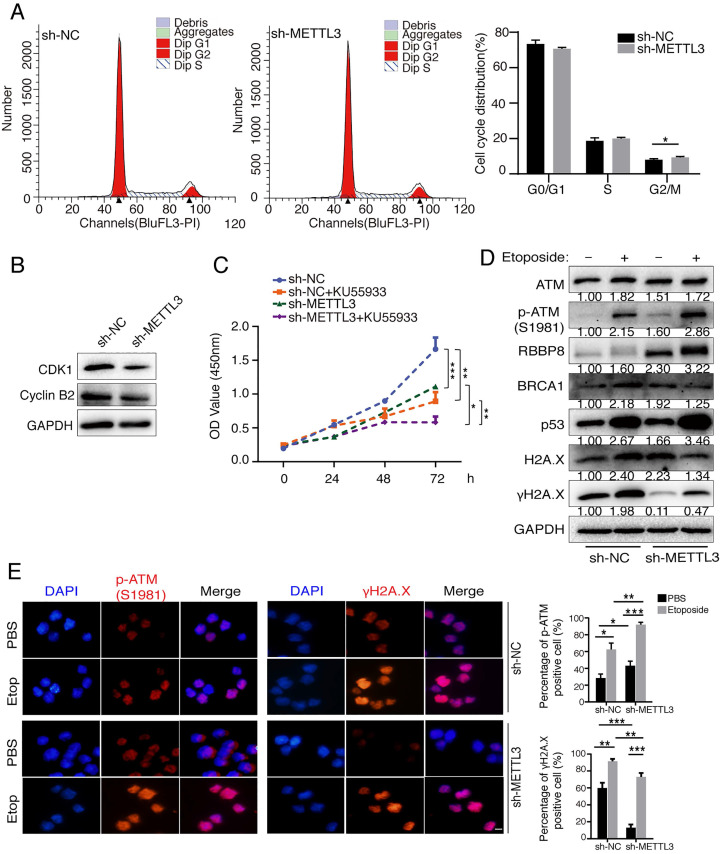
** METTL3 promotes cell cycle progression, cell proliferation and involves in the DNA damage response. (A and B**) SGC7901 cells (sh-NC and sh-METTL3) were cultured to a predetermined time, and cell cycle was assayed by flow cytometry. Data are representative of three independent experiments (A). Western blot was used to test the indicated proteins (B). **(C)** SGC7901 cells (sh-NC and sh-METTL3) were cultured to a predetermined time in the presence or absence of the ATM inhibitor KU55933 (10 μM), and then CCK8 assay was performed. Mean ± SD of three independent experiments. **(D and E)** BJAB cells (sh-NC and sh-METTL3) were cultured in the presence or absence of etoposide (10 μM) for 8 h. Western blot analysis of indicated proteins (D). Immunofluorescence analysis of DNA damage response related proteins (E). Scale bar: 20 μm. *p < 0.05, **p < 0.01, ***p < 0.001.

Previous studies have identified that METTL3 facilitated tumor progression via an m6A-dependent mechanism 29, 30. To further verify the oncogenic role of METTL3, we inhibited METTL3 in gastric cancer cells SGC7901 to detect its effects on cell cycle and cell proliferation. Knockdown of METTL3 resulted in an increase in G2/M phase (Figure 3A). Cyclin B2 contributes to the G2/M phase transition by activating CDK1 kinase, and cyclin B2 inhibition induces cell cycle arrest 31. We found that inhibition of endogenous METTL3 significantly reduced the expression levels of CDK1 and cyclin B2, implying that G2/M phase block may be attributed to the downregulation of CDK1 and cyclin B2 (Figure 3B). Inhibition of METTL3 also decreased tumor cell proliferation by means of CCK8 assay (Figure 3C). Next, we pre-treated cells with specific ATM inhibitor KU55933 32, 33, and CCK8 assay revealed that KU55933 significantly reduced cell proliferation. In addition, inhibition of METTL3 in combination with KU55933 significantly inhibited cell proliferation, comparing to sh-METTL3 and KU55933 only group (Figure 3C). These results suggested that simultaneous inhibition of METTL3 and ATM could be used as a therapeutic strategy to inhibit tumor cells proliferation.

Next, we investigated the effect of METTL3 on the ATM downstream signaling. Expression levels of ATM, p-ATM, RBBP8, BRCA1, H2A.X and p53 were significantly increased, whereas the levels of γH2A.X (reflecting DNA damage signal) was significantly decreased after the inhibition of METTL3 expression without etoposide treatment (Figure 3D, lane 3 vs lane 1). Following the addition of etoposide (to induce a DNA damage response in cells 34), the expression levels of ATM, p-ATM, RBBP8, BRCA1, p53 and H2A.X were increased (Figure 3D, lane 2 vs lane 1). Overall, the DNA damage response was stronger in sh-NC group comparing to sh-METTL3 group, as reflected by the more significant increase in p-ATM, RBBP8, BRCA1, and p53 (Figure 3D, lane 2/1 vs lane 4/3). We also examined the effects of sh-METTL3 on p-ATM (S1981) and γH2A.X levels under the etoposide treatment by immunofluorescence. sh-METTL3 increased p-ATM, whereas decreased γH2A.X levels (Figure 3E). In other words, when the m6A modification system is compromised (for instance, METTL3 inhibition), the DNA damage response is becoming “weaker”, and the DNA damage sites are more abundant in cells.

### YTHDF1 or YTHDF2 binds to *ATM* mRNA and regulates its downstream signaling

**Figure 4:**
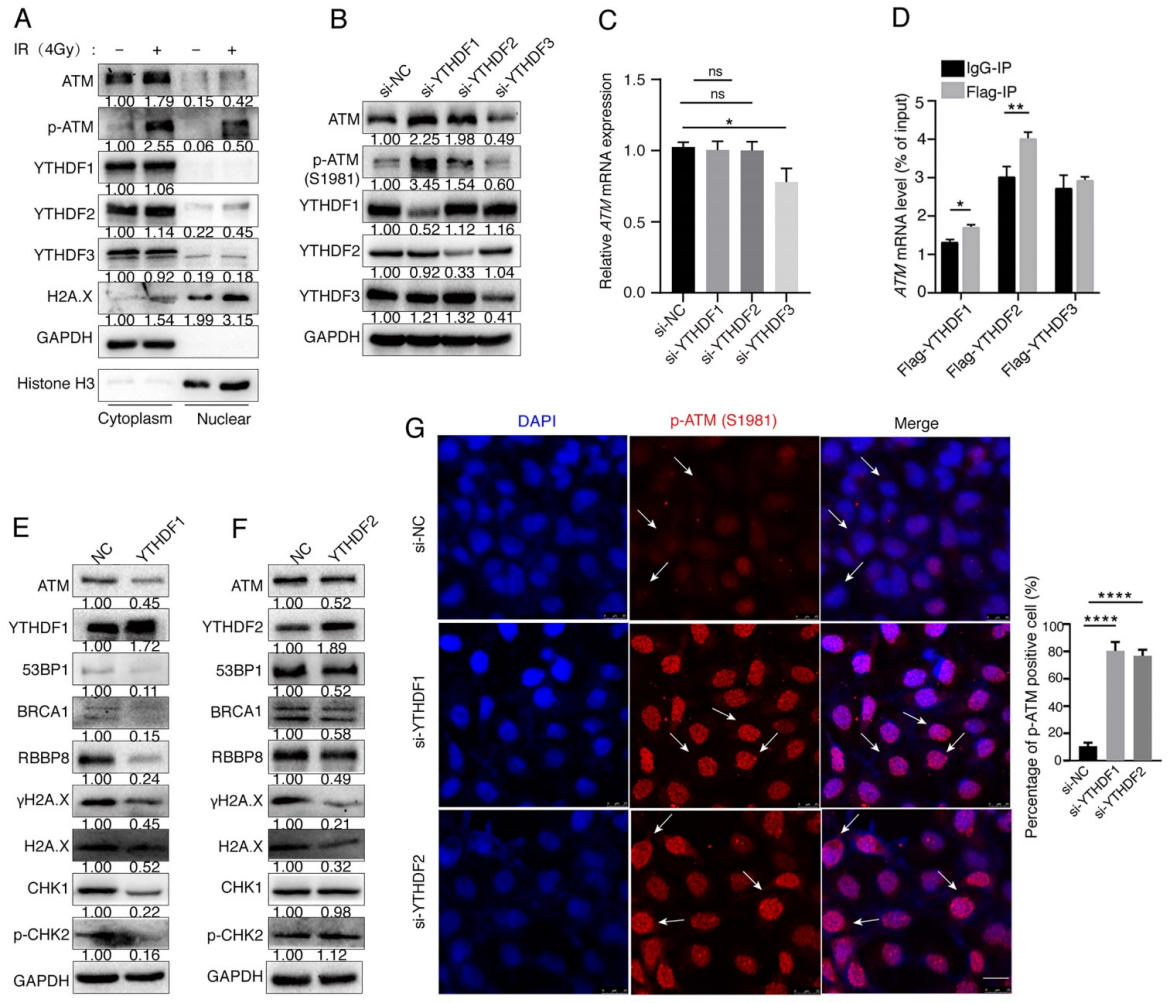
** YTHDFs bind to ATM mRNA and regulate its downstream signaling. (A)** SGC7901 cells were irradiated with ionizing radiation (4Gy) for 30 minutes, and then cytoplasmic and nuclear proteins were isolated. **(B and C)** SGC7901 cells were transfected with indicated siRNAs for 48 h. Western blot (B) and RT-qPCR (C) were used to assay the indicated molecules. **(D)** RIP-qPCR. SGC7901 cells were transfected with Flag-tagged YTHDF1, YTHDF2, or YTHDF3 expression plasmids for 48 h. Cell lysates were immuoprecipitated with anti-Flag beads, and then RNAs were isolated from the immunoprecipitates, and used for RT-qPCR to detect ATM mRNA levels. Mean ± SD of three independent experiments. Two-tailed unpaired t test. *p < 0.05, **p < 0.01. **(E and F)** SGC7901 cells were transfected with the YTHDF1 or YTHDF2 expression vectors for 48 h, respectively, and the cellular proteins were isolated for Western blot assay. **(G)** SGC7901 cells were transfected with siRNAs (si-YTHDF1 or siYTHDF2) for 48 h and immunofluorescence assay was performed to detect the fluorescence expression of p-ATM (Ser1981). Scale bar: 25 μm. ****p < 0.0001.

The m6A “readers” participate in post-transcriptional regulation by binding directly to the m6A modification sites of target mRNAs. m6A “readers” YTH family proteins are mainly distributed in the cytoplasm, suggesting that their functions may be related to RNA in the cytoplasm and play a role in the translation and degradation of RNA 35-37. To investigate the distribution of ATM protein and m6A reader proteins during the DNA damage, we irradiated SGC7901 gastric cancer cells with ionizing radiation (4Gy) for 30 min, and found that ionizing radiation-induced DNA damage resulted in increased expression of ATM, p-ATM, and H2A.X. YTHDF1, YTHDF2 and YTHDF3 proteins were mainly localized in the cytoplasm. The nuclear localizations of H2A.X, p-ATM, and YTHDF2 were increased upon ionizing radiation (Figure 4A). We used siRNAs to suppress the expression of endogenous YTHDF1 or YTHDF2, and noticed that the expression levels of ATM and p-ATM proteins were significantly increased. However, inhibition of YTHDF3 decreased the expression levels of ATM and p-ATM proteins (Figure 4B). Inhibition of YTHDF1 or YTHDF2 did not alter the *ATM* mRNA expression levels as detected by RT-qPCR, whereas si-YTHDF3 decreased *ATM* mRNA levels (Figure 4C).

To explore the mechanism of how m6A modifications regulate ATM expression, we hypothesized that m6A readers YTHDF proteins could bind to *ATM* mRNA and regulate mRNA translation. We transfected Flag-tagged YTHDF1, YTHDF2, and YTHDF3 expression vectors into cells, and found that *ATM* mRNA was enriched with Flag-YTHDF1 and Flag-YTHDF2, but not Flag-YTHDF3, suggesting that YTHDF1 and YTHDF2 could bind to *ATM* mRNA (Figure 4D). We further investigated whether YTHDF1 or YTHDF2 could affect the expression of downstream molecules of the ATM signaling. Eexpression of YTHDF1 inhibited the expression of ATM, 53BP1, BRCA1, RBBP8, γH2A.X, Chk1, and p-Chk2 (Figure 4E). Meanwhile, YTHDF2 reduced the expression levels of ATM, 53BP1, BRCA1, RBBP8, γH2A.X, but not the CHK1 and p-CHK2 (Figure 4F). In addition, immunofluorescence experiments confirmed that inhibition of YTHDF1 and YTHDF2 upregulated p-ATM expressions (Figure 4G). These results suggested that YTHDF1 and YTHDF2 are involved in the regulation of ATM downstream signaling.

### YTHDF1 suppresses ATM expression in the post-transcriptional levels

**Figure 5:**
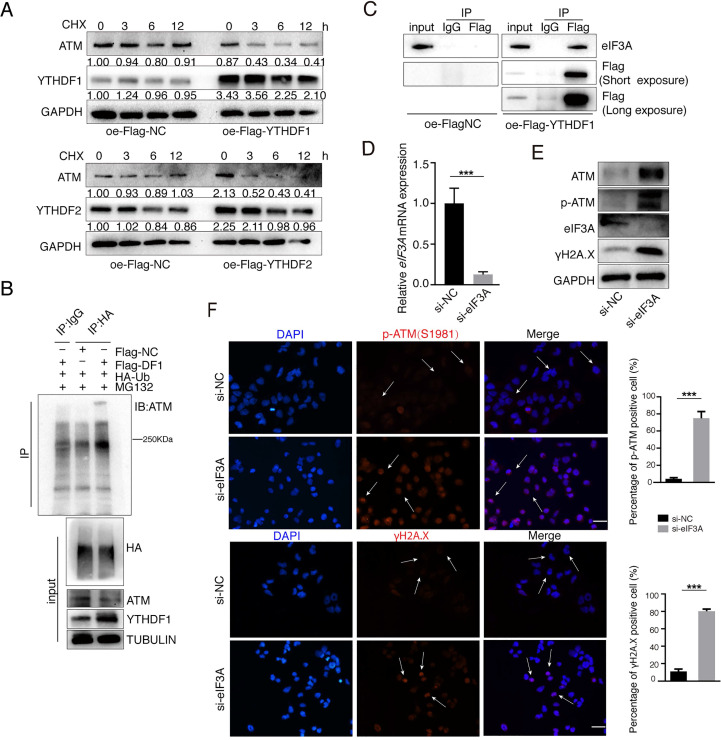
** YTHDFs suppress ATM expression in the post-transcriptional levels. (A)** SGC7901 cells were transfected with Flag-NC or Flag-YTHDF1 or Flag-YTHDF2 for 36 h, followed by CHX treatment (25 μg/ml) for indicated times. Cellular proteins were collected and Western blot was performed. **(B)** HEK293 cells were transfected with indicated plasmids (Flag-NC, Flag-YTHDF1 and HA-Ubiquitin), and cells were treated with MG132 (25 μM) for 4 h before harvest. Lysates were immunoprecipitated with anti-HA antibodies and then immunoblotted with anti-ATM antibodies. **(C)** HEK293 cells were transfected with Flag-NC or Flag-YTHDF1 for 48 h, then cell lysates were subject to immunoprecipitation with anti-Flag antibodies. The immunoprecipitates were subsequently blotted with indicated antibodies. **(D and E)** SGC7901 cells were transfected with indicated siRNAs for 48 h. RT-qPCR **(D)** and Western blot **(E)** were used to assay the indicated molecules. Mean ± SD of three independent experiments. Two-tailed unpaired t test. **(F)** SGC7901 cells were transfected with indicated siRNAs for 48 h. Immunofluorescence assays were performed to detect the fluorescent expression of p-ATM (Ser1981) and γH2A.X. Scale bar: 50 μm. ***p < 0.001.

We further explored how YTHDFs influence ATM in the post-transcriptional levels. We used Cycloheximide (CHX, 25 μg/ml) to prevent protein synthesis and examined the stability of ATM protein. Both YTHDF1 and YTHDF2 significantly reduced the stability of ATM protein (Figure 5A). We co-transfected the Flag-YTHDF1 and HA-Ubiquitin plasmids into cells and found that overexpression of YTHDF1 increased ubiquitination levels of ATM proteins (Figure 5B). eIF3A is also a m6A “reader”, and study has suggested that YTHDF1 regulates the translation process by interacting with eIF3A or eIF3B 38. Co-IP analysis showed that YTHDF1 is interacting with eIF3A protein (Figure 5C), and we speculate that YTHDF1 binds to *ATM* mRNA while recruiting eIF3A to regulate *ATM* mRNA translation. Inhibition of endogenous eIF3A with siRNA increased the expression levels of ATM proteins and is involved in ATM downstream signaling (Figure 5D, E). Immunofluorescence confirmed that inhibition of eIF3A upregulated p-ATM and γH2A.X levels (Figure 5F). These results suggested m6A readers YTHDF1/eIF3A could decrease ATM protein stability.

In addition, we also found that inhibition of YTHDF1 decreased the G0/G1 phase and increased S-phase in cancer cells (Supplementary Figure 1A). p21 is known to inhibit the kinase activity of cyclin A/CDK1, 2, leading to cell cycle inhibition into S phase 39, 40 Mechanistically, we showed that inhibition of YTHDF1 significantly decreased p21 expression, and increased CDK1, CDK2 expressions, whereas cyclin B2 had no change (Supplementary Figure 1B). Inhibition of YTHDF1 suppressed tumor cell proliferation. In addition, inhibition of YTHDF1 in combination with ATM inhibitor KU55933 significantly inhibited cell proliferation, comparing to si-YTHDF1 and KU55933 only group (Supplementary Figure 1C). These results suggested YTHDF1 promotes cancer cell proliferation and cell cycle.

## Discussion

RNA m6A modifications play important roles in a variety of biological processes. Xiang *et al*. revealed a novel role for RNA m6A modification in promoting cellular resistance to UV damage, and identified a potential new pathway involving METTL3, FTO, m6A RNA modification, and Pol κ in the early UV-induced DNA damage response 22. However, the regulatory mechanisms of m6A modification on the ATM signaling are still unclear. In the current study, we mainly focused on the m6A modifications of ATM and the impact on ATM downstream signaling.

**Figure 6:**
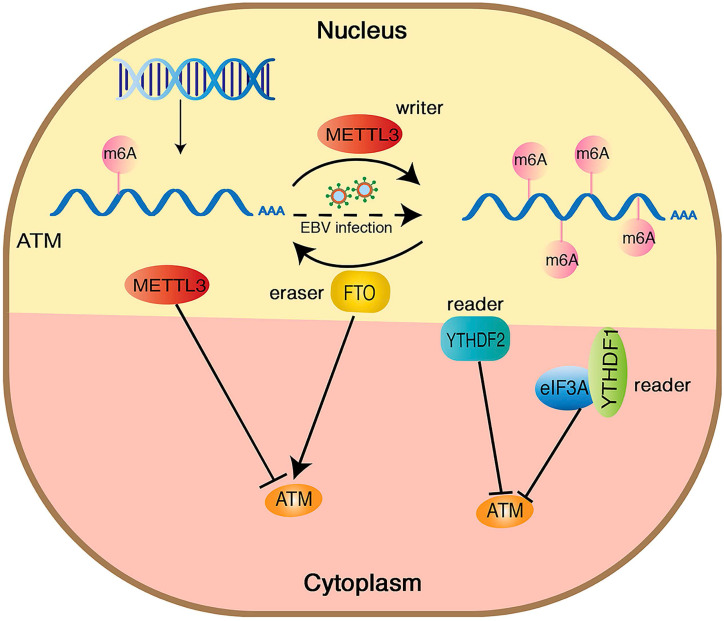
Hypothetical model of the mechanism how m6A modification regulates ATM expression and downstream signaling. METTL3 increases the m6A modification of ATM and inhibits ATM expression and mRNA stability, whereas FTO functions oppositely. YTHDF1/2 bind to the m6A sites and recruit eIF3A, leading ATM degradation.

ATM kinase is a major regulator of the DNA damage response, and the activation of ATM upon DNA damage involves multiple cascade reactions, including acetylation 41, UFMylation 42 and destabilization of linker histone H1.2 32. In this study, we confirmed the presence of m6A modifications on ATM mRNA by MeRIP. Inhibition of METTL3 (an m6A “writer”) upregulated ATM expression levels and increased ATM mRNA stability. Meanwhile, inhibition of FTO (an m6A “eraser”) downregulated ATM expression levels, suggesting that m6A modifications could dynamically regulate ATM expression levels (Figure 6).

METTL3 is an important component of the m6A methyltransferase complex, and it also acts as an oncogene in the process of tumorigenesis 43. Luo *et al*. previously reported that silencing METTL3 significantly suppressed melanoma cell proliferation and colony formation 44. Wang *et al*. demonstrated that METTL3 promoted gastric cancer cell proliferation and metastasis 30. Yang *et al*. found that METTL3 promotes the progression of gastric cancer via targeting the MYC pathway 45. Consistent with these results, we showed that METTL3 accelerate cell cycle progression and cell proliferation in gastric cancer cells; and inhibition of METTL3 in combination with specific ATM inhibitor KU55933 further inhibited cancer cell proliferation, comparing to sh-METTL3 and KU55933 only group. In addition, we found that METTL3 also modulated the ATM downstream signaling: when the expression of METTL3 was inhibited, the ATM signaling (the DNA damage response) was less evident, and the DNA damage sites were more abundant, indicating an important role of m6A modification in modulating ATM signaling.

As m6A “readers”, YTH family proteins serve as important binding proteins for m6A modifications to regulate RNA metabolism, including splicing, translation, and degradation 37, 38. We used RIP assay to reveal that YTHDF1 and YTHDF2 could bind to *ATM* mRNA. Zhuang *et al.* had demonstrated that YTHDF1 regulated the translation of m6A-modified *Robo3.1* mRNA. Knockdown or mutation of YTHDF1 resulted in a dramatic reduction of Robo3.1 protein without affecting *Robo3.1* mRNA level 46. Consistent with their study, we found that YTHDF1 or YTHDF2 increased ATM protein degradation but did not affect *ATM* mRNA expressions. We further confirmed that overexpression of YTHDF1 promoted ATM degradation via ubiquitination. Additionally, YTHDF1 is involved in regulating the mRNA translation by interacting with eIF3A and eIF3B translation initiation factors 15, 38. Our co-IP experiments revealed that YTHDF1 interacted with eIF3A. Silencing of eIF3A could increase ATM protein levels. We speculated that YTHDF1 recruits eIF3A to promoting ATM protein degradation. However, the specific mechanism of how YTHDF1 recruits eIF3A to regulate ATM expression has yet to be determined in the future.

In summary, we have revealed for the first time the presence of m6A modifications in *ATM* mRNAs. m6A modification regulates *ATM* mRNA metabolism and ATM downstream signaling, which illustrates the importance of m6A modification-related molecules for being used as therapeutic targets in DNA damage-related diseases.

## Supplementary Material

Supplementary figure and tables.
