# Supplementary material for: N6-methyladenosine regulates ATM expression and downstream signaling
Source: J Cancer. 2021 Oct 17;12(23):7041–51. doi: 10.7150/jca.64061 (PMC8558655; doi:10.7150/jca.64061)
Supplement: Supplementary file 1 — Supplementary figure and tables. [file jcav12p7041s1.pdf]

Supplementary Table S1. Sequences of siRNA and shRNA

Name	sequences
si-NC	sense 5’-UUCUCCGAACGUGUCACGUTT-3’
	antisense 5’-ACGUGACACGUUCGGAGAATT-3’
si-FTO	sense 5’-GGUGCUCCGUGAAGUUAAATT-3’
	antisense 5’-UUUAACUUCACGGAGCACCTT-3’
si-YTHDF1	sense 5’-GGAUACAGUUCAUGACAAUTT-3’
	antisense 5’-AUUGUCAUGAACUGUAUCCTT-3’
si-YTHDF2	sense 5’-CUGCUUAUCGUUCCAUGAATT-3’
	antisense 5’-UUCAUGGAACGAUAAGCAGTT-3’
si-YTHDF3	sense 5’-CAAUUCAAGGGACACUCAATT-3’
	antisense 5’-UUGAGUGUCCCUUGAAUUGTT-3’
si-eIF3A	5’- CAGTTGATGGCAAATTACT -3’
LV3-sh-NC	5’-TTCTCCGAACGTGTCACGT-3’
LV3-sh-Mettl3	5’-GGGCCCAAGTGCAAGAATTCT-3’

Supplementary Table S2. Primer sequences for qRT-PCR

Name	sequences
ACTIN	Forward 5’-GAGCTACGAGCTGCCTGACG-3’
	Reverse 5’-GTAGTTTCGTGGATGCCACAG-3’
ATM	Forward 5’-ATCTGCTGCCGTCAACTAGAA-3’
	Reverse 5’-GATCTCGAATCAGGCGCTTAAA-3’
YTHDF1	Forward 5’-ACCTGTCCAGCTATTACCCG-3’
	Reverse 5’-TGGTGAGGTATGGAATCGGAG-3’
YTHDF2	Forward 5’-AGCCCACTTCCTACCAGATG-3’
	Reverse 5’-TGAGAACTGTTATTTCCCCATGC-3’
YTHDF3	Forward 5’-TCAGAGTAACAGCTATCCACCA-3’
	Reverse 5’-GGTTGTCAGATATGGCATAGGCT-3’
eIF3A	Forward 5’-GCCGGAAAATGCCCTCAAAC-3’
	Reverse 5’- TGGTTCGTGTATCTTTTGCCAT-3’
METTL3	Forward 5’-TTGTCTCCAACCTTCCGTAGT-3’
	Reverse 5’- CCAGATCAGAGAGGTGGTGTAG-3’

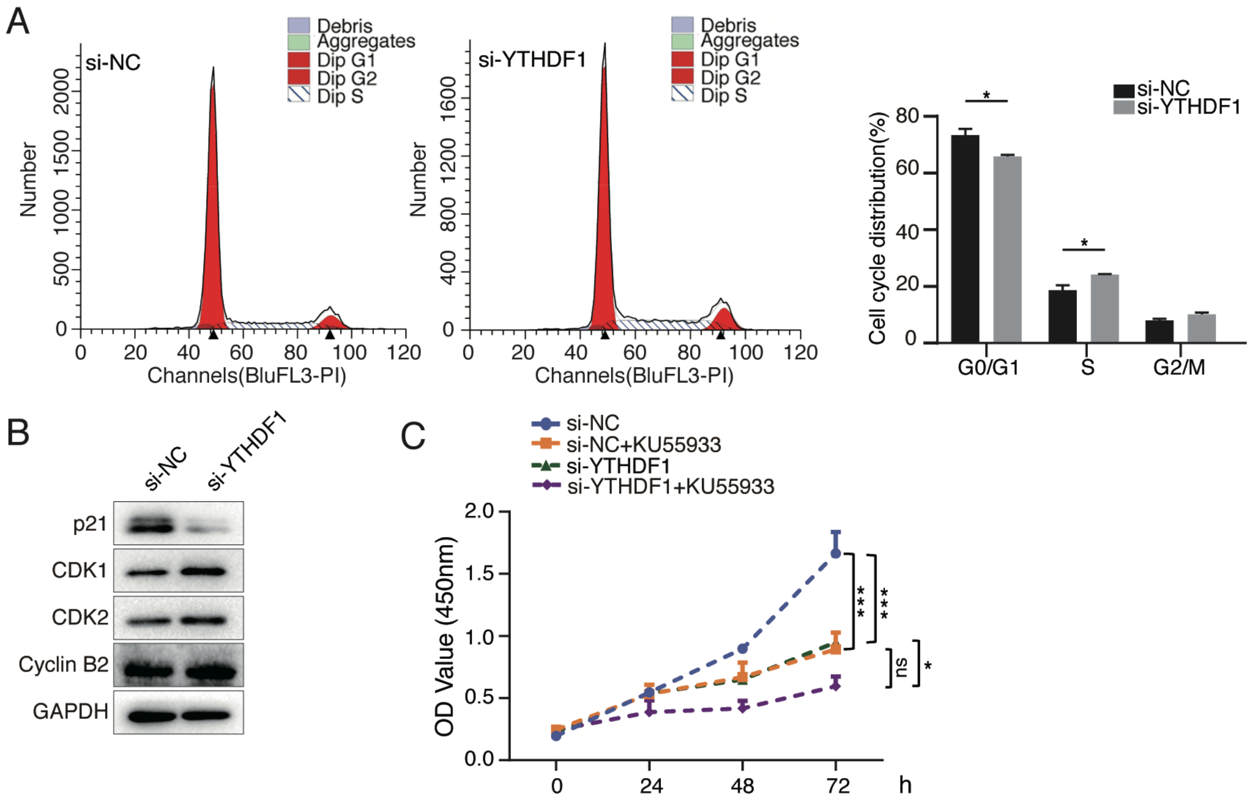

Supplementary Figure 1. (A and B) SGC7901 cells were transfected with siRNAs (siNC and siYTHDF1) and cultured to a predetermined time, and cell cycle was assayed by flow cytometry. Western blot was used to test the indicated proteins. (C) SGC7901 cells (sh-NC and sh-YTHDF1) were cultured to a predetermined time in the presence or absence of the ATM inhibitor KU55933 (10 μM), and then CCK8 assay was performed. Mean ± SD of three independent experiments. Two-tailed unpaired t test. *$p < 0.05$, ***$p < 0.001$.
